# Cholera in Lagos State, Nigeria: a One Health perspective on outbreaks and public health interventions

**DOI:** 10.3389/fpubh.2026.1755792

**Published:** 2026-03-31

**Authors:** Malik Olatunde Oduoye, Khadija Motunrayo Musa, Komal Zulfiqar, Osama Ahmad, Muhammad Owais, Zainab Riasat, Ruhma Sehar, Muhammad Hammad Nazrul Islam, Eram Shahzadi, Sana Rasheed, Muhammad Usman Haider

**Affiliations:** 1 Department of Research, The Medical Research Circle (MedReC), Goma, Democratic Republic of Congo; 2 Department of Nursing Science, University of Nigeria, Nsukka, Enugu, Nigeria; 3 Department of Medicine, Allama Iqbal Medical College, Lahore, Pakistan; 4 Department of General Surgery, Khyber Teaching Hospital, MTI, Peshawar, Pakistan; 5 Department of Medicine, Liaquat University of Medical and Health Sciences, Jamshoro, Pakistan; 6 Department of Medicine, Quaid-e-Azam Medical College, Bahawalpur, Pakistan; 7 College of Medical Sciences, Ahmadu Bello University, Zaria, Kaduna, Nigeria; 8 Department of Surgery, Faisalabad Medical University, Faisalabad, Pakistan; 9 Department of Medicine and Surgery, Jinnah Sindh Medical University, Karachi, Pakistan; 10 Department of Internal Medicine, Geisinger Health System, Danville, PA, United States

**Keywords:** cholera, interventions, Lagos, Nigeria, One Health, outbreak, public health

## Abstract

**Background:**

Cholera continues to pose a significant public health challenge in Nigeria, particularly in Lagos, where rapid urbanization, inadequate sanitation, and climatic variability converge to heighten vulnerability. This study reviews recent cholera outbreaks in Lagos through a One Health perspective, emphasizing their implications, intersectoral drivers, and existing response gaps.

**Methods:**

A systematic review was conducted using 48 articles published between 2013 and 2024, retrieved from PubMed, Google Scholar, and official sources such as the Center for Disease Control and Prevention (CDC) and the World Health Organization (WHO). Search terms included “Cholera,” “Lagos,” “Nigeria,” and “One Health.” Eligible studies addressed human, animal, and environmental determinants of cholera transmission and control.

**Results:**

The review identified 401 cholera cases and 21 deaths in Lagos State in 2024, and three reported cases in 2025. Cholera imposes substantial physical, psychological, and economic burdens. Physically, approximately one in 10 Lagosians may experience adverse health effects, ranging from dehydration to multi-organ complications. Psychologically, fear of infection and crisis conditions contribute to stress, anxiety, and stigma. Economically, cholera generates high direct and indirect costs across households, the health system, government, and the wider economy. Recurring outbreaks are strongly associated with inadequate Water, Sanitation, and Hygiene (WASH) infrastructure, high population density, environmental contamination, and limited laboratory capacity.

**Conclusion:**

Effective public health interventions are essential to mitigate cholera outbreaks in Lagos, preventing increased transmission, mortality, and economic disruption. Persistent outbreaks highlight the absence of a coordinated One Health policy in the state. Integrating human, animal, and environmental surveillance within a unified framework is critical to achieving Nigeria's cholera elimination targets by 2030.

## Introduction

Cholera poses a significant threat to public health in developing countries where access to safe water and sanitation facilities is limited. Cholera, an acute diarrheal illness caused by the bacterium *Vibrio cholerae*, is characterized by watery stools and has been documented since ancient times (1). Throughout the 19th and 20th centuries, the disease spread globally beyond Asia on seven separate occasions, each referred to as a cholera pandemic (1). The first cholera pandemic was recorded in 1817, originating in South Asia and later spreading globally in 1829, 1852, 1863, 1881, 1889, and 1961 (2). The seventh cholera pandemic, which began in 1961, reached the African continent around 1971 (2). However, unlike in parts of Asia, the pandemic form has not persisted in Nigeria. Instead, cholera has remained endemic, characterized by periodic but contained outbreaks.

A five-decade review reported that major epidemics occurred in 1991, 2010, and 2021, reflecting episodic resurgence rather than continuous transmission (3). More recent investigations indicate that current Nigerian outbreaks are driven primarily by multidrug-resistant atypical El Tor and non-O1/non-O139 *V cholerae* strains, whereas the typical El Tor lineage associated with the early phase of the seventh pandemic is no longer epidemic or endemic in the country (4). Approximately three to five million people worldwide are affected by cholera each year, and over 100,000 cases result in death (5).

In 31 states across 107 local government areas (LGAs), Nigeria has reported 53 deaths and 1,528 suspected cases of cholera (6). These figures were disclosed by the Director General of the Nigeria Center for Disease Control and Prevention (NCDC), who also noted that the case fatality rate, since the start of 2024, was 3.5 per cent (6). Several of the fatalities were attributed to individuals presenting in an advanced stage of dehydration, and were unable to revive them due to the severity of their condition. In some cases, these patients were even brought in already dead (7). In Lagos State, 29 people have unfortunately lost their lives due to cholera, while 30 others are currently being treated in hospitals (6). Regrettably, the northeast region of Nigeria was plagued by a decade-long onslaught of Boko Haram attacks, which resulted in an estimated 20,000 deaths and the displacement of 2.6 million people (8). Many of these displaced individuals were left without adequate access to food and clean water (8).

Regarding the potential part played by flies and contaminated surfaces, commonly referred to as fomites, in spreading the illness. Houseflies could potentially act as carriers of the bacteria, mechanically disseminating cholera (5). *Vibrio cholerae* infection can cause a range of health outcomes, from asymptomatic colonization to severe diarrhea. Untreated or unboiled drinking water is a major risk factor for cholera, while soap use is linked to reduced infection likelihood (9). In foodborne cholera outbreaks, risk factors include consuming certain foods like rice products, vegetables, or fruits. In areas with sporadic cholera, most cases are associated with shellfish consumption (10). Host-pathogen interactions, such as blood group O, are linked to severe cholera because Individuals with blood group O exhibit increased intestinal binding of cholera toxin, leading to more severe fluid loss (11). Other contributing factors include hypochlorhydria and retinol deficiency (11).

Engaging in open defecation and utilizing shared sanitation facilities are associated with an elevated risk of cholera transmission. Although narrow-mouthed water containers are typically associated with reduced bacterial contamination, leaving these containers uncovered or repeatedly handled by hands or utensils can negate this protective effect and increase the risk of bacterial transmission (12). Climate change poses a substantial threat to Water, Sanitation, and Hygiene (WASH) infrastructure and services due to the intrinsic link between water and climate. The ongoing effects of climate change have already and will continue to impact water availability in rivers, lakes, and underground aquifers, alter precipitation patterns, and escalate the frequency and severity of extreme weather events such as floods and droughts (13). Microbial contamination of water has been demonstrated to be correlated with open defecation practices, with rainfall facilitating the runoff of waste from open defecation sites into watercourses (14, 15).

Common symptoms of cholera include abdominal pain, cramps, stomach rumbling, and vomiting, particularly in the early stages. Severe diarrhea can result in significant fluid and electrolyte loss, although fever is rare (1). Cholera is commonly associated with the presence of fecal matter and bile during the initial stages of the disease. Nevertheless, the defining characteristic of severe cholera, also known as “cholera gravis,” is the production of copious amounts of watery stool containing mucous flecks, which is commonly referred to as “rice-water” stool (16). This type of stool typically has a fishy odor and is usually without any sensation of straining. In extreme cases, adults can produce up to 1 L of stool per hour (1).

To address the challenges of cholera outbreaks in Lagos State, an integrated One Health approach represents a comprehensive strategy compared with traditional single-sector or outbreak-driven responses, as it integrates human health surveillance, environmental monitoring, and WASH interventions (17). However, despite several studies published on cholera in Nigeria, there is limited awareness about the implications and One Health lessons of cholera in Lagos, Nigeria. It is feared that a new pandemic is most likely to emerge from a convergence of multiple factors, including antimicrobial resistance, zoonotic spillover, fragile health systems weakened by budget constraints, and insufficient global coordination, all of which interact and intensify within today's highly interconnected world (17).

One Health's relevance is particularly evident in the context of cholera, a waterborne disease that significantly affects global health, especially in Nigeria's health infrastructure. Since the first cholera outbreak in 1970, multiple factors such as inadequate access to potable water, poor sanitary conditions, environmental and climatic changes, rapid urbanization, increasing population growth, and inadequate emergency or public health responses have kept the country vulnerable (18).

This study explores the recent cholera outbreak in Lagos State, including its health implications and public health interventions. The implication gap entails integrated, multi-sectoral cholera prevention strategies in Lagos, Nigeria, where rapid urbanization, inadequate WASH infrastructure, and environmental vulnerability converge, while the public health interventions would cover assessing how human, animal, and environmental health factors intersect in driving and mitigating cholera transmission in the state.

## Methodology

### Systematic review design

A systematic review was conducted in accordance with the Preferred Reporting Items for Systematic Reviews and Meta-Analyses (PRISMA) 2020 guidelines (19).

### Study area

The review is focused on Lagos State because it is the most populous, industrious, and cholera-prone state in Southwestern Nigeria. Lagos State is one of the southwestern states in Nigeria, with a 2025 estimated population of 17, 156, 400 (20). Lagos State acts as the commercial hub for the country and is inhabited by a substantial population of more than 20 million individuals (20).

### Search strategy

**Figure 1:**
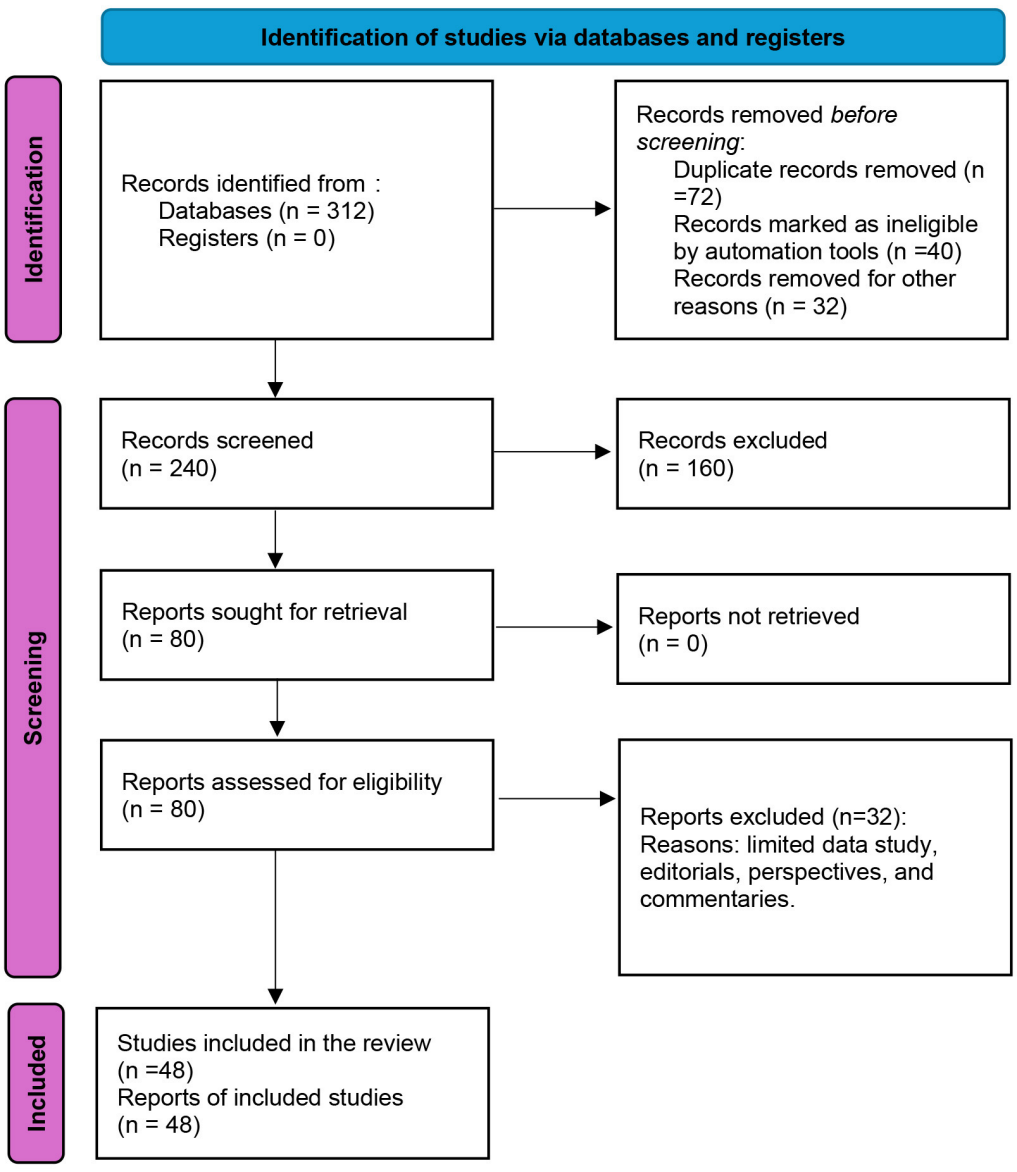
PRISMA chart for study selection.

The screening process followed PRISMA 2020 guidelines for systematic reviews, with titles and abstracts independently screened by two reviewers for eligibility (see Figure 1). Extraction of relevant studies that employed relevant articles, selected from past and present studies through a literature search from electronic databases, including PubMed, Google Scholar, and the Center for Disease Control and Prevention (CDC) and the World Health Organization (WHO) websites, on the subject matter from 2013 to 2024, using keywords such as “Cholera,” “Lagos,” “Nigeria,” “Outbreak,” and “One Health,” combined through Boolean operators. Duplicate records were removed manually using Microsoft Excel before title and abstract screening. Two independent reviewers screened articles for eligibility, with disagreements resolved by consensus. The initial database search yielded 312 records. After removal of duplicates (72), 240 articles were screened by title and abstract. Following full-text assessment, 48 studies were included in the final synthesis (Figure 1). A total of 48 articles published between 2013 and 2024 were reviewed.

#### Inclusion criteria

Review articles, meta-analyses, case reports, and literature reviews, government and international health reports written in English were included on the rationale that they merit the scope and aim, and Medical Subject Headings (MeSH) of this study, to get scientific data about this study. Data from the included studies were manually extracted by the authors through careful reading and note-taking.

Key points such as study location, relevance to the One Health framework, main findings, and policy recommendations were recorded. While no formal quality scoring tool was used, studies were appraised for relevance, peer-reviewed status, and credibility. Studies were included if they: focused on cholera outbreaks in Nigeria or sub-Saharan Africa with relevance to the One Health framework; provided quantitative or qualitative data on cholera prevalence, determinants, control strategies, or public health interventions; and were published in English.

#### Exclusion criteria

Non-English papers, news articles, editorials, and perspectives were excluded due to their limited data contribution. In addition, studies such as commentaries or non-peer-reviewed reports without original data were not considered.

#### Data synthesis

Extracted data were synthesized thematically across epidemiological trends, environmental determinants, WASH infrastructure, and intervention strategies. A qualitative narrative approach was used due to the heterogeneity of study designs.

#### Data extraction

Extracted variables included geographical location, study population, number of patients, clinical presentation, interventions or treatment strategies, and mortality outcomes. Data were grouped and synthesized by region (Southwest, Northern, and National-level studies) and thematically analyzed to highlight One Health dimensions (human, animal, and environmental factors).

## Results

### Study selection

Following database searching and screening, a total of 48 studies published between 2013 and 2025 met the eligibility criteria and were included in the final synthesis (Figure 1). These studies reported epidemiological data, environmental determinants, WASH-related risk factors, and intervention strategies relevant to cholera outbreaks in Lagos State (7, 9–16, 18, 21–61).

### Cholera outbreaks in Lagos State

The extracted data indicate recurrent cholera outbreaks in Lagos over the study period. In 2024, Lagos State recorded 401 confirmed cholera cases and 21 deaths, while three cases were reported in early 2025 (22–28). Table 1 shows major Cholera Outbreaks in Lagos State (2010–2025).

**Table 1:** Major cholera outbreaks in Lagos State (2010–2025).

Year	Suspected case	Confirmed case	Death	Case fatality rate (CFR)	References
2010	3,000+	–	120	3.8%	(26, 28)
2017	27	–	2	7.4%	(24)
2020–2021	78	–	5	6.4%	(23)
2024	401	43	21	5.4%	(26)
2025 (Jan–Mar)	1,149 (nationwide)	–	28 (nationwide)	2.4%	(26)
2025 (Lagos only)	3	–	0	0%	(26)

Earlier outbreaks were documented across multiple Local Government Areas, with higher case concentrations consistently observed in densely populated informal settlements and waterfront communities (21–24). Several studies reported seasonal clustering of cases, with peaks typically occurring during periods of heavy rainfall and flooding (21, 22). For example, a total of 22,931 cases and 2,945 fatalities were documented in Nigeria (8). From 1991 to 2018, an alarming 321,148 cholera cases, including 18,644 deaths, were reported throughout Nigeria (3). The number of cases of cholera during the epidemic in Lagos State, between 2004 and 2014, was 4,060, with 20 deaths due to cholera (15).

**Figure 2:**
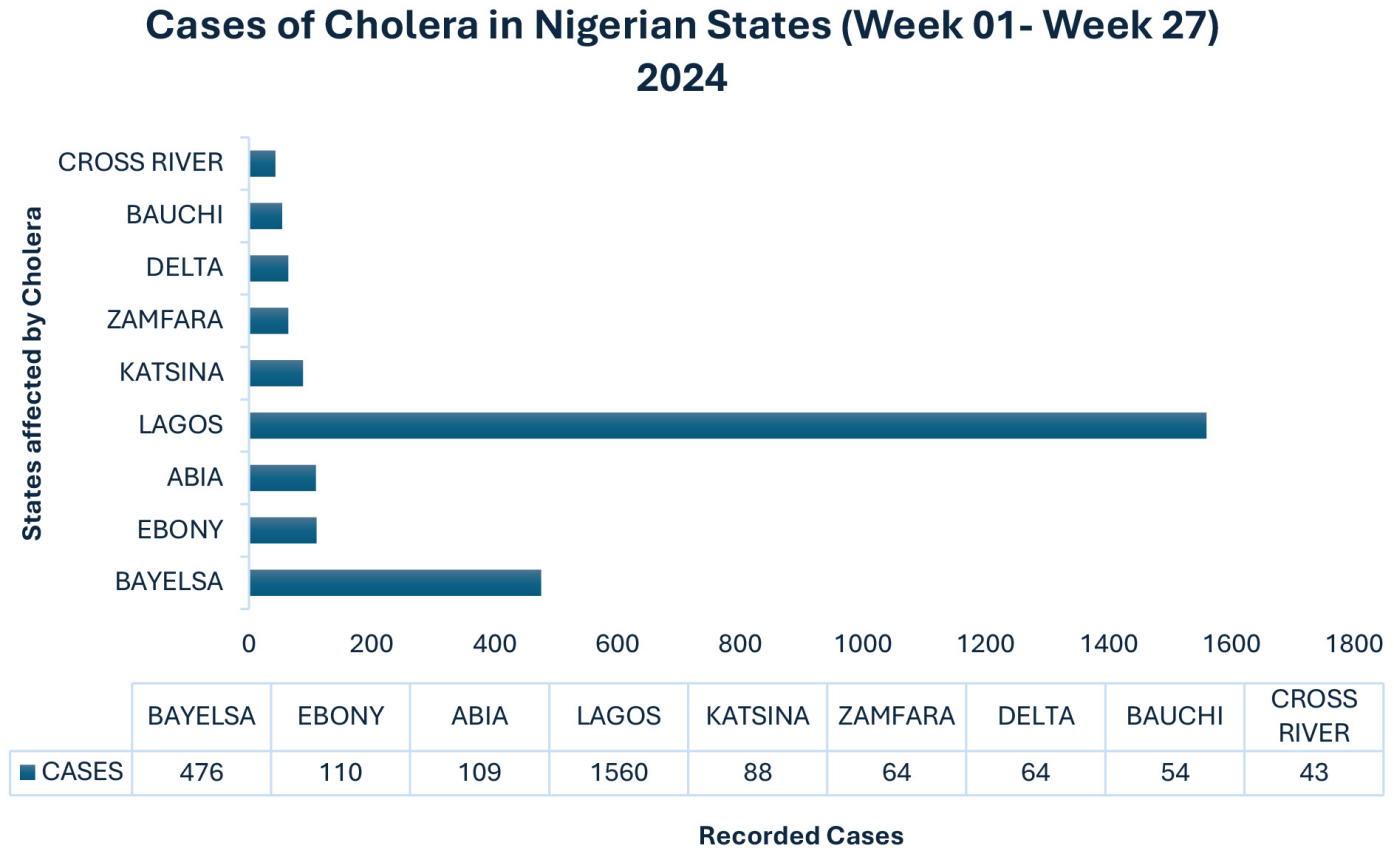
This figure depicts the prevalence of cholera cases in Nigeria in 2024. Source: World Health Organization (61) and Clemens et al. (16).

Despite substantial investments in reinforcing its primary healthcare infrastructure, the city reported 27 probable instances of cholera and two deaths in July 2017 (23). In Lagos, during the period between October 12th, 2020, and October 25th, 2021, a total of 78 cases were reported, out of which five resulted in fatalities (24). The graph displayed in Figure 2 depicts the prevalence of cholera cases in Nigeria in 2024.

### Implications of cholera in Lagos

#### Physical implications

Extracted evidence indicates that most individuals infected with cholera in Lagos State experience mild or no symptoms; however, approximately one in 10 affected persons develop significant physical complications (27). Reported early clinical manifestations include acute watery diarrhea, excessive thirst, dry mouth, reduced urine output, and sunken eyes. Disease progression is associated with electrolyte disturbances resulting in muscle cramps, generalized weakness, cardiac complications (including myocarditis, pericarditis, and arrhythmias), and acute kidney injury (28, 29).

Further reported outcomes include hypovolemia leading to hypotension and tachycardia, with fatal outcomes documented in severe cases. Respiratory complications such as pneumonia and acute respiratory distress syndrome, as well as neurological manifestations including seizures, encephalopathy, and Guillain-Barré syndrome, were also identified among affected Lagos residents (30, 31).

Additional contributing factors reported in Lagos include extreme weather events and conflict-related disruptions (32). Cultural practices such as mountaintop burials, open defecation, weddings, and funerals were identified as exposure-related factors. Studies also reported that preference for traditional medicine and avoidance of formal healthcare facilities were associated with delayed treatment and non-adherence to cholera management protocols (33).

#### Mental health implications

Included studies documented psychological effects associated with cholera outbreaks in Lagos. Reported outcomes included stress and anxiety linked to fear of infection and crisis conditions. Exposure to severe illness and high mortality was associated with trauma and post-traumatic stress disorder (34). Stigmatization of affected individuals contributed to social isolation and depressive symptoms (35, 36). High workloads during outbreaks were also associated with burnout among healthcare workers (37). Some reports noted increased community cohesion during outbreak responses (38).

#### Economic costs and implications

Cholera outbreaks in Lagos were reported to impose substantial direct and indirect economic costs across households, healthcare systems, government institutions, and the wider economy (26, 39). Direct medical expenditures included outpatient and inpatient care, intravenous fluids, antibiotics, laboratory investigations, oral rehydration salts, and case management, borne by both health facilities and households (26).

Direct non-medical costs included transportation to healthcare facilities, funeral and burial expenses, and household purchases of water treatment and hygiene supplies (26, 40). Indirect costs included productivity losses due to patient and caregiver absenteeism, reduced income, school absenteeism, and decreased productivity within affected communities (26, 39, 40).

Public-sector expenditures included surveillance, laboratory testing, emergency response operations, risk communication, WASH infrastructure repair, community mobilization, and vaccine deployment (39). Cholera outbreaks were also reported to negatively affect tourism, trade, and business confidence in Lagos State, with recurrent epidemics diverting public resources from essential services (3).

Although Lagos-specific cost-of-illness studies were limited, outbreak reports from national agencies provided estimates of household financial burden. One study reported that affected households in Lagos incurred an average expenditure of N=77,633.78, approximately 52 USD per cholera episode, including both direct and indirect costs (40). These findings are summarized in Table 2.

**Table 2:** Implications of cholera outbreaks in Lagos State.

Dimension	Key finding	Example	References
Physical	Acute watery diarrhea, dehydration, and multi-organ complications	1 in 10 Lagosians affected	(16, 24, 56)
Mental	Stress, anxiety, stigma, PTSD, and burnout among health workers	Fear of contracting cholera	(34, 39)
Economic	Direct medical costs, productivity losses, and household expenditures	(N=77,633.78)~52 USD per episode	(49, 52)
One Health	Human, animal, and environmental drivers	WASH gaps, flooding, insecurity	(18, 22)

#### One Health implications

Only a limited number of included studies reported One Health–related findings in Lagos. Available evidence described interactions between human health, environmental conditions, and socio-behavioral factors in cholera transmission (18). Studies applying the One Health framework emphasized integrated consideration of population health, environmental sanitation, and community practices in outbreak contexts (18).

## Discussion

This present study situates cholera outbreaks in Lagos State within the broader regional context of recurrent cholera transmission across sub-Saharan Africa, while emphasizing Lagos-specific vulnerabilities. Globally, cholera remains prevalent in South Asia, sub-Saharan Africa, and Latin America, with epidemic regions reporting case fatality rates exceeding 3%, compared with ≤ 1% in endemic settings (1, 5). Several African countries, including Nigeria, Zimbabwe, Angola, and Sudan, have historically reported epidemic case fatality rates ranging from 3.3 to 4.3%, with even higher rates documented during major outbreaks in Haiti and Madagascar (5). Over four million cholera cases were reported across Africa between 1970 and 2017, reflecting sustained endemicity in multiple regions (21).

More recently, between January 2022 and July 2024, over 399,000 cases and 7,023 deaths were recorded across the continent, with the Democratic Republic of the Congo, Ethiopia, Malawi, Mozambique, and Zimbabwe accounting for more than 70% of cases (22). These figures likely underestimate the true burden due to incomplete surveillance (41–43). Within Nigeria, cholera remains a persistent public health challenge. Although the March 2025, the NCDC report documented only three cases in Lagos State alongside sporadic infections in neighboring southwestern states, this represents a shift from earlier large-scale epidemics observed in 2010, 2018, and 2021 (26). Nationally, the 2021 outbreak was the largest on record, exceeding 111,000 suspected cases, and nearly 20,000 cases were reported by December 2024, with further increases documented in early 2025 (43). Despite some reductions in mortality over time, Nigeria's estimated case fatality rate of 3.2%−3.7% in 2024 remains well above the WHO target of less than 1%, underscoring persistent weaknesses in case management and outbreak response systems (3).

In Lagos State, cholera transmission is sustained by inadequate water and sanitation infrastructure, frequent flooding, rapid urbanization, climate variability, and population displacement. These interacting drivers create persistent exposure pathways, particularly in peri-urban settlements and flood-prone communities (39). While recent patterns suggest a transition from widespread epidemics toward more localized transmission, the continued emergence of new cases highlights ongoing structural vulnerabilities a7gnd the risk of resurgence if preventive investments are not maintained (18).

Nigeria's Integrated Disease Surveillance and Response system (IDSR) serves as the primary framework for outbreak detection and management; however, its effectiveness in Lagos is constrained by limited community-level surveillance capacity, delayed reporting due to poor connectivity, inadequate laboratory infrastructure, logistical challenges, and data quality issues arising from under-reporting and restricted diagnostic confirmation (44, 46). Although Emergency Operations Centers and rapid response teams are mobilized during outbreaks, interventions remain largely reactive. Shortages of trained personnel, diagnostic kits, and essential supplies such as oral rehydration salts further compromise timely control efforts (46).

Laboratory capacity remains a critical bottleneck. Many cholera-prone areas face shortages of qualified personnel, inconsistent supply chains, challenges in specimen transport and storage, and weak reporting systems, limiting the use of culture-based diagnostics and hindering strain surveillance (41). Insecurity and access constraints in vulnerable regions also disrupt preparedness activities, delay deployment of supplies, and impair case management, affecting all pillars of outbreak response (41, 42). High population density in displacement camps further complicates containment efforts (47).

Financial constraints significantly impede effective cholera control. In 2021, only 11% of the required WASH funding and 17% of the health-sector funding under Nigeria's Humanitarian Response Plan had been secured, delaying outbreak detection and response while increasing morbidity and mortality (48, 49). These funding gaps also restrict the capacity to monitor circulating *Vibrio cholerae* strains and adapt interventions, including vaccines and treatment protocols, as epidemiological patterns evolve (50).

Marked regional disparities in WASH infrastructure persist across Nigeria, with rural populations representing nearly half of the national population experiencing substantially lower access to improved sanitation than urban residents (51). Such inequities place millions at elevated risk of cholera and other waterborne diseases (42). In Lagos, socioeconomic disadvantage, overcrowding, unsafe water access, and environmental degradation converge to amplify transmission risk, with extreme weather events and conflict acting as additional catalysts (32).

Conflict-related disruptions further exacerbate vulnerability by causing displacement, interrupting essential services, and undermining livelihoods. Descriptive application of a self-controlled case-series framework suggests temporal associations between environmental exposures such as flooding and spikes in cholera incidence in Lagos. Broader regional evidence indicates that cholera risk increases by approximately 3.6-fold in Nigeria and 2.6-fold in the Democratic Republic of the Congo during periods of violence (50). Delays in outbreak detection median of 29 days from symptom onset to identification in conflict-affected settings, substantially increase the likelihood of uncontrolled transmission (47).

These findings underscore the importance of a One Health approach that integrates human health surveillance with environmental monitoring, WASH programming, and multisectoral coordination. Sharing WASH, environmental, and vector-control data across agencies can accelerate outbreak detection and response, while coordinated multisectoral strategies have been associated with measurable reductions in response delays globally (33, 47). Alignment with the Global Roadmap to End Cholera highlights the necessity of sustained political commitment, strengthened surveillance, resilient WASH infrastructure, and cross-sector collaboration to reduce cholera-related mortality and prevent future outbreaks in Lagos State and Nigeria more broadly (42).

### Public health interventions

Based on the findings, the Lagos State government should enhance public health interventions, such as adequate surveillance and reporting mechanisms, in Lagos State to detect cholera cases promptly by implementing specific strategies, such as vaccination and WASH programs, in high-risk areas to prevent the spread of cholera (42). Instances of cholera have been effectively decreased in Bangladesh and Haiti through the implementation of integrated WASH programs in conjunction with health education and vaccination efforts (43). These examples depict how well-coordinated measures can be used to control cholera epidemics in Lagos State.

Meanwhile, the Lagos State government should also engage with local communities to spread more awareness about cholera, its spread, and prevention. Community education regarding the use of all these precautionary measures, practicing oral rehydration, and early medical consultation is equally important and should be rigorously used as a preventive tool, especially in high-risk populations (44). Additionally, to monitor and control the animal sources of cholera in Lagos, the government should strengthen coordination with animal health services through joint surveillance of livestock, aquatic animals, and water sources. Integrating data sharing, environmental testing, and hygiene training under a One Health approach would enable early detection and more effective prevention of cholera transmission (45).

Another intervention is geographic Information Systems (GIS) and remote sensing technologies, which can help to map and predict cholera spread in Lagos, enabling targeted interventions (46). The Lagos State government can adopt the early warning systems (EWS) and rapid response to control the scourge of cholera in the state, as this system has been utilized across the world, including the Cholera Map (Mobile App) in Bangladesh, climate and weather-driven EWS in Africa, and an electronic early warning and response network (EWARN) in Somalia and the East Mediterranean region (47–49). The basic reliance of all EWS is on surveillance data. These systems incorporate alert mechanisms, threshold-based indicators, and community reporting networks. Once an alert is triggered (suspected case of cholera), response procedures are enacted swiftly, involving the rapid mobilization of healthcare resources, distribution of clean water and sanitation supplies, and public health communication campaigns to inform and protect at-risk populations (47, 50).

The Lagos State government can prevent cholera through a comprehensive approach that includes routine immunization, ensuring access to safe food and clean water, improving sanitation and personal hygiene, and strengthening community engagement and health education to promote preventive practices and early reporting of suspected cases (47). The two main vaccines currently used in endemic areas, Dukoral (Contains killed whole cells of *V. cholerae* O1 and a recombinant B subunit of cholera toxin) and Shanchol (Contains killed whole cells of *V. cholerae* O1 and O139), have efficacies of 85%−90% (50).

It is proven that oral cholera vaccines (OCVs) are safe, but lower in effectiveness, with immunity waning rapidly, requiring frequent boosters (48). However, healthcare providers in Lagos State should utilize Vaxchora, an FDA-authorized single-dose live attenuated oral cholera vaccine, which provides 90% protection against severe diarrheal symptoms 10 days post-immunization and 80% protection after 3 months, primarily used for travelers (51).

Moreover, investing in infrastructure to provide clean WASH facilities is fundamental for cholera prevention and management in Lagos State (57). Specific measures, like digging latrines and boreholes in populations at risk, can drastically lower the spread of illness. It is essential to implement safe storage, handwashing procedures, and water purification. In community trials, low-cost tactics like solar-powered water purifiers and handwashing stations are successful. Since drinking potable water can eradicate cholera outbreaks, it is imperative to guarantee that all Lagosians have access to it (62).

The Lagos State government can tackle the economic cost implications of cholera through case data by obtaining confirmed or suspected case numbers for Lagos (7, 58). Or per-case cost by application of a cost range of N=30,000–N=150,000 (USD 20–100 per case), reflecting variations in care intensity and income loss; Lagos figures are likely higher due to urban living costs. The Lagos State government should aggregate estimation through summing direct, indirect, and public-sector costs, with response costs estimated at 10%−30% of household costs (26). There should be an increase in the funding for cholera prevention and response programs, which will, in turn, increase the capacity of laboratories for prompt cholera diagnosis and surveillance (59, 63).

There is a need for international health authorities, such as the WHO and the United Nations (UN), to support the development of global cholera control strategies. They should provide more funding and technical assistance for cholera control programs in Lagos State (57, 60). Furthermore, the Lagos State government should foster international collaboration on cholera control to promote the research and development of new and effective cholera vaccines, which is a need of the time (61). Table 3 shows recommended public health interventions.

**Table 3:** Recommended public health interventions.

Intervention area	Specific action	Expected outcome	References
WASH infrastructure	Expand access to clean water, improve sanitation	Reduced transmission	(22, 49)
Surveillance	Strengthen IDSR, integrate One Health monitoring	Early detection	(26, 61)
Community engagement	Hygiene education, stigma reduction	Increased prevention	(9, 39)
Technology	GIS, remote sensing, EWS	Improved prediction	(53, 54)
Policy alignment	WHO Roadmap 2030, national strategies	Sustainable elimination	(22, 61)

**Figure 3:**
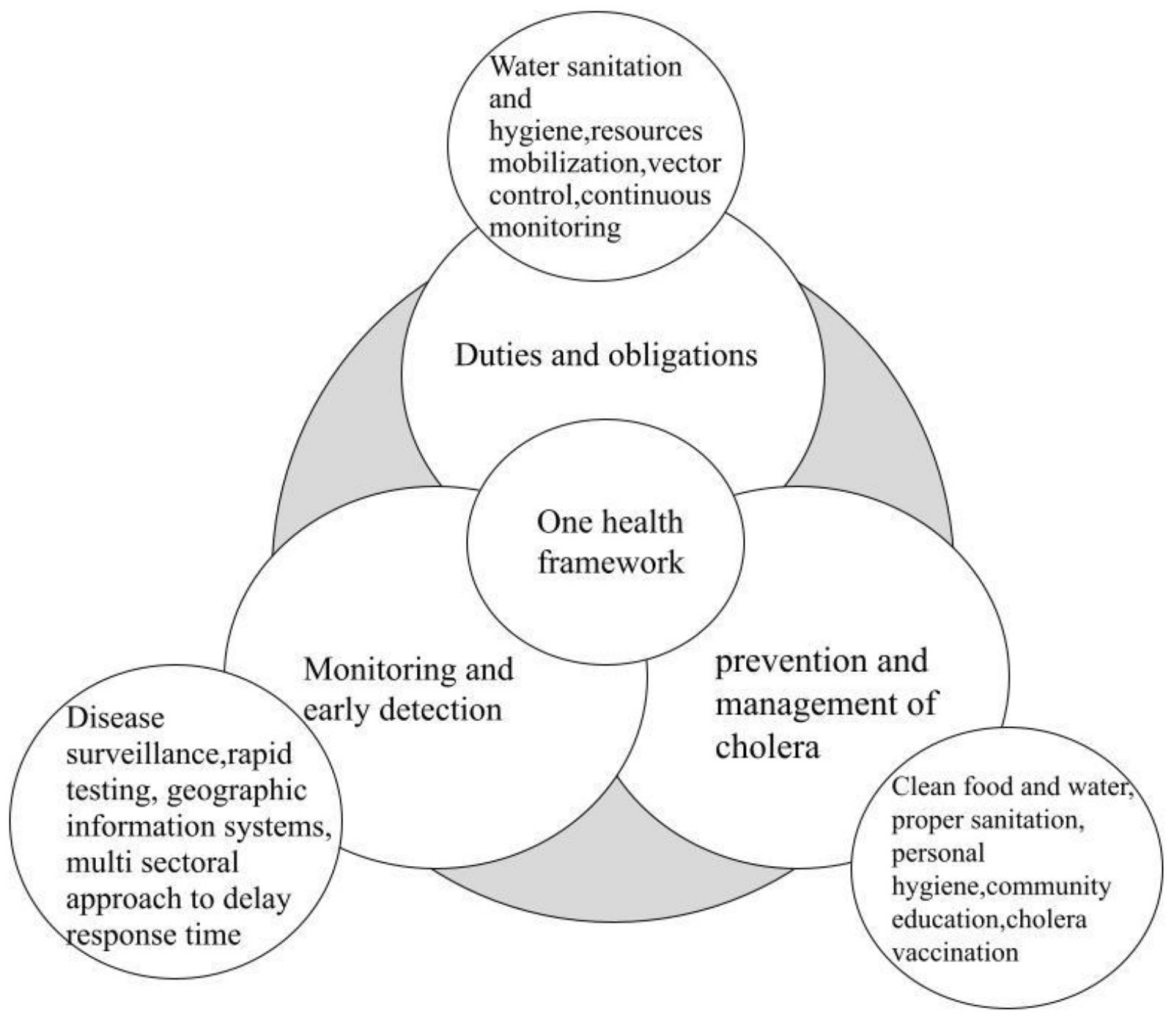
This figure illustrates a health framework designed to combat cholera cases. Adapted from Ihekweazu et al. (18) and WHO (61).

Effective cholera control in Lagos requires cross-border collaboration and integration with national surveillance systems and regional partnerships coordinated through the Global Task Force on Cholera Control (GTFCC) framework, which promotes multisectoral coordination, standardized data sharing, and joint outbreak preparedness across West African countries (61). The GTFCC recommends that countries increase their capacity to contain small outbreaks and suggests methods and systems to detect and contain potential outbreaks (61). Establishing rapid response teams for robust disease surveillance and rapid testing is a key contributory factor to limiting a cholera outbreak (64). Surveillance systems collect real-time data by continuously tracking reported cases, identifying hotspots, and monitoring early trends. Figure 3 illustrates a health framework designed to combat cholera cases.

### Study limitations

This study has several limitations. As a systematic review, it relied exclusively on secondary data sources without primary field data collection, which may limit the contextual depth and accuracy of findings specific to Lagos State. The exclusion of non-English publications and gray literature may have introduced publication and selection bias, potentially omitting relevant local health reports and surveillance data. In addition, no formal quality appraisal tool was applied to the included studies, which may affect the robustness and reliability of the synthesized evidence.

The heterogeneity of study designs, outcome measures, and reporting standards further precluded quantitative synthesis, such as meta-analysis or formal trend analysis, thereby limiting the ability to estimate effect sizes or assess temporal patterns. Moreover, Lagos-specific data were limited for certain periods, and inconsistencies in surveillance systems may have resulted in underreporting of cholera cases.

Although a One Health perspective was adopted, integration of data across human, animal, and environmental health sectors was constrained by limited cross-sectoral evidence. The absence of stakeholder perspectives, including healthcare workers, WASH practitioners, and affected community members, may also reduce the practical applicability of the recommendations. Nevertheless, this review provides a consolidated overview of key drivers, vulnerabilities, and response patterns relevant to cholera control in Lagos State.

Future research should prioritize primary data collection, incorporate standardized quality assessment tools, include gray literature, and strengthen One Health data integration through collaboration with veterinary and environmental agencies. Engaging local stakeholders and conducting comparative analyses across Nigerian states would further enhance the evidence base and policy relevance of subsequent studies.

## Conclusion

This review underscores the recurrent nature of cholera outbreaks in Lagos State, largely driven by persistent deficiencies in WASH infrastructure, alongside environmental and climatic vulnerabilities. Strengthening public health interventions is therefore essential to reduce transmission and cholera-related morbidity and mortality in the state. Such efforts should prioritize sustained community engagement and risk communication, the strategic use of innovative tools such as geographic information systems and early warning systems, and strengthened national and international collaboration. Aligning these interventions within a coordinated One Health framework will be critical to supporting long-term cholera control and advancing Nigeria's progress toward global cholera elimination targets.

## Data Availability

The original contributions presented in the study are included in the article/supplementary material, further inquiries can be directed to the corresponding author.
